# Optimization of an organic photovoltaic device via modulation of thickness of photoactive and optical spacer layers

**DOI:** 10.1186/1556-276X-9-460

**Published:** 2014-09-02

**Authors:** Qi Li, Won Jung Yoon, Heongkyu Ju

**Affiliations:** 1 Department of Bionano Technology, Gachon University, Gyeonggi-do 461-701, South Korea; 2 Department of Chemical & Bio Engineering, Gachon University, Gyeonggi-do 461-701, South Korea; 3 Department of Nano-Physics, Gachon University, Gyeonggi-do, 461-701, South Korea; 4 Neuroscience Institute, Gil Hospital, Incheon, 405-760, South Korea

**Keywords:** Solar cell, Power conversion efficiency, Optical spacer, Bulk-heterojunction, Series resistance

## Abstract

We examine the modulation effects of thicknesses of both a photoactive layer (a bulk-heterojunction (BHJ) of poly(3-hexylthiophene) and [6,6]-phenyl-C61-butyric acid methyl ester (P3HT:PCBM)) and an optical spacer of a transparent metal oxide, for power conversion efficiency optimization of organic photovoltaic devices. The redistribution of the optical intensity at the photoactive layer via the thickness modulation of both layers is taken into account, to produce three-dimensional (3D) plots as a function of both layer thicknesses of 0 to 400 nm range (5 nm step), for the device efficiency optimization. The modulation pattern of absorption is produced in the 3D plot as scanning the thicknesses of both layers as a result of modulation of interference between incoming and reflected light, which can be secured by changing the effective optical path length between two electrodes of a photovoltaic device. It is also seen that the case of inserting the spacer of the higher refractive index demands finer adjustment of the spacer layer thickness to achieve the optimum device efficiency.

In addition, the series resistance of the photoactive layer of the thickness range of 0 to 70 nm is taken into account to provide the 3D plots as a function of the scanned thicknesses of both layers. Inclusion of the series resistance of the photoactive layer, which is also the function of its thickness, in the simulation, indicates that the series resistance can influence qualitatively the dependence of power conversion efficiency (PCE) on the thicknesses of both layers. We also find that minimization of series resistance, e.g., by device annealing, allows not only the relevant voltage to increase but also the optimum thickness of the photoactive layer to increase, leading to more absorption of light.

## Background

The past several decades have witnessed the rapid development of an organic photovoltaic device. Enormous efforts have been devoted to the improvement of organic photovoltaic efficiencies [1-6] with the recent report of power conversion efficiency (PCE) of about 12% [7-11]. Particularly, it is known that bulk-heterojunction (BHJ)-based organic photovoltaic devices exhibit advantages such as the low cost, its flexibility, its light weight, and the solution processibility for its fabrication.

However, the limited electron-hole transport characteristics of a BHJ photoactive layer do not simply encourage the active layer elongation that can benefit from the expansion of absorption region, because the elongated transport distance increases the rate of recombination of photo-generated charge carriers [12]. To enhance the device performance, various device structures that employ optical spacers [13,14], photonic crystals [15], and localized surface plasmon resonance are proposed. More interestingly, optical spacers inserted between two electrodes of a photovoltaic device have been studied to redistribute light intensity profiles across the photoactive layers where absorption occurs to maximize the absorption. This redistribution is attributed to optical interference changes, resulting from the changes in an optical path length between two metal electrodes that collect photo-generated carriers.

Previous studies on optical spacers, which show enhancement of absorption at the photoactive layer via the insertion of optical spacers, however, have used a number of discrete values of spacer thicknesses over the limited range for given thicknesses of the photoactive layers. In addition, one may not simply estimate the device performance, i.e., short-circuit current as thicknesses of both photoactive and spacer layers are modulated due to the following two reasons: first, both thickness modulations (two different materials) complicate the redistribution of light intensity at the photoactive layer via the modulation of the effective optical path length; and second, the photoactive layer thickness also governs the series resistance of the device, which consists of both the active layer bulk resistance and its contact resistance with the electrode [16-19]. For example, the increase in the photoactive layer thickness that is expected to expand the absorption region may not simply result in the enhancement of the PCE due to the resistance change.

In this paper, we theoretically investigate the performance efficiency of a BHJ organic photovoltaic film comprising a blend of semiconducting polymer (poly(3-hexylthiophene): P3HT) and fullerene derivative ([6,6]-phenyl-C61-butyric acid methyl ester: PCBM). This photoactive film is sandwiched between the charge separating electrodes, i.e., the hole collecting electrode of poly(3,4-ethyl-enedioxythiophene):poly(styrene-sulfonate): PEDOT:PSS-indium tin oxide (ITO) glass, and the electron collecting electrode of aluminum (Al). Insertion of a metal oxide optical spacer between the Al electrode and the photoactive layer (P3HT:PCBM) is considered to optimize the optical intensity distribution over the photoactive region.

By scanning the thicknesses of both the photoactive and optical spacer layers from 0 to 400 nm with a 5 nm step, we provide a three-dimensional (3D) plot of the short-circuit current (*J*_*sc*_) at the photoactive layer, taking into account the expansion of absorption region and light intensity redistribution at the layer. The modulation behavior of *J*_*sc*_ is observed along the scanned thickness of each layer over the entire range scanned in the 3D graph as a result of optical field redistribution caused by the effective path-length change between two electrodes. The modulation behavior becomes more faded out at larger thickness of the photoactive layer. Optical spacers of different refractive index materials, i.e., ZnO and TiO_2_, are also examined, revealing that the higher index spacer demands the finer adjustment of the spacer layer thickness for device efficiency optimization.

The series resistance (*R*) which is known to vary with the photoactive layer thickness [16] is used to normalize *J*_*sc*_ to represent the device performance (*η* = *J*_*sc*_/*R*). Inclusion of such thickness-dependent resistance in the 3D plots of *η* produces the qualitative change in the 3D plot shape of the normalized *J*_*sc*_, implying that the thickness-dependent resistance causes the qualitative change in the dependence of device performance on both thicknesses of the active layer and the spacer. It is therefore seen that the reduction of series resistance permits not only the relevant voltage to increase but also the optimum thickness of the photoactive layer to increase leading to more absorption that takes place.

## Methods

There are two commonly used methods to calculate distributions of optical fields in a multilayer structure of thin films where interference between incident and reflected light occurs. One of them is by using a transfer matrix method based on matrices of optical propagation during the constituent media and those of interface with field boundary conditions [20]. The other one is the finite difference time domain (FDTD) method, which has recently been widely used for solving Maxwell's equations for a given optical system of a complex structure.

**Figure 1:**
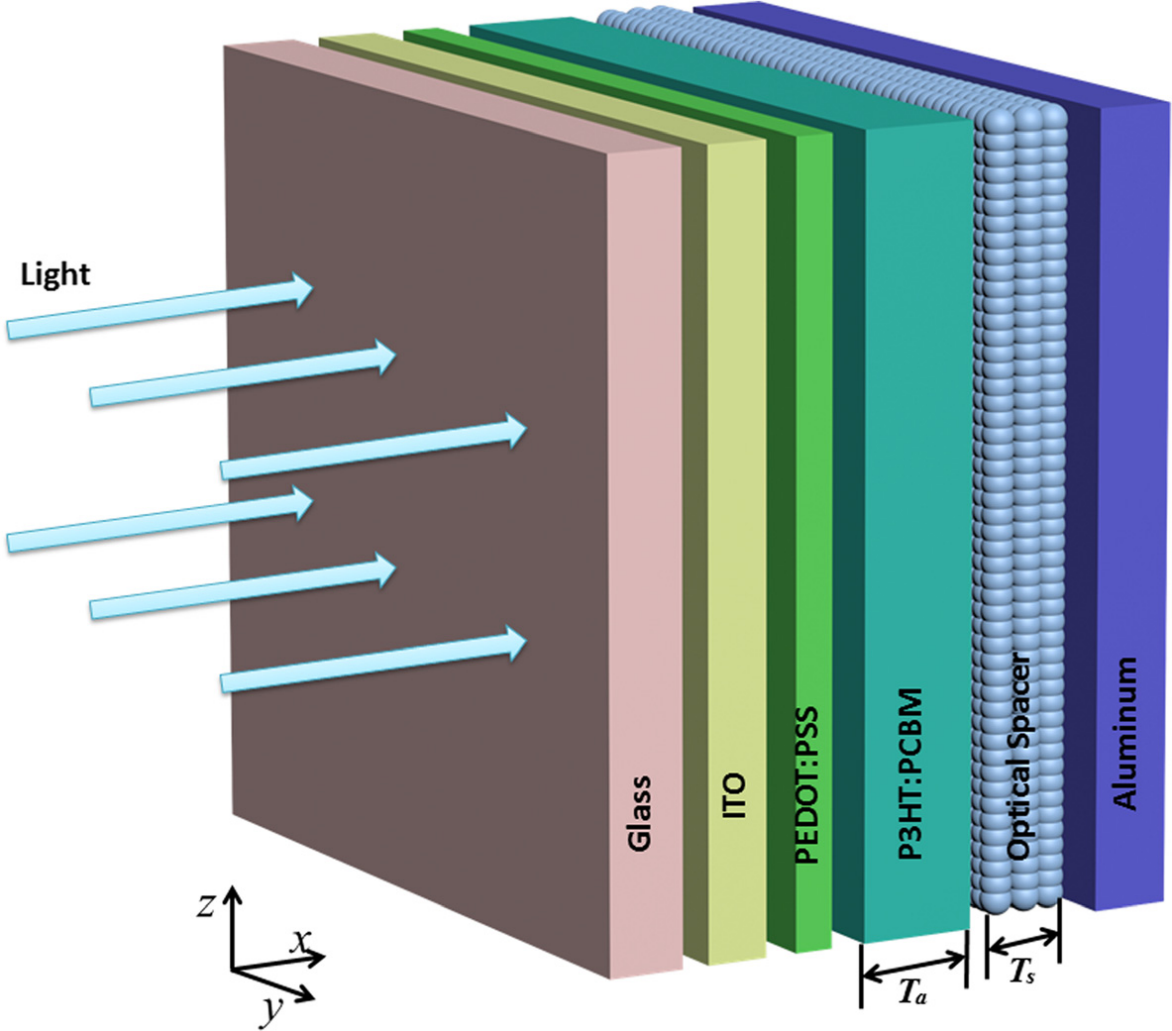
Schematic of structure of an organic photovoltaic device considered.

A FDTD method relies on numerical calculation with discrete meshes in both space and time. A discrete mesh made of so-called Yee cells is used to describe the electromagnetic fields and structural materials of interest. Maxwell's equations are solved discretely in time, where the time step used is dominated by a mesh size through the speed of light. We use the software called FDTD Solutions (Lumerical Solutions, Inc., Vancouver, BC, Canada) for calculation of optical field distribution within the photovoltaic cell of interest. This calculation includes the interference effects between optical fields propagating in the forward direction and those in the backward direction within the photovoltaic device.Scanning of thicknesses of device constituent layers changes a condition for optical interference between incident and reflected light, thus allowing the optimum condition to be achieved for optimum field distribution for energy harvest efficiency. Figure 1 shows schematic of the organic photovoltaic structure considered in the simulation, where we scan the thicknesses of both the active layer (P3HT:PCBM) and the optical spacer, while keeping constant the thicknesses of the other layers, i.e., an ITO and PEDOT:PSS layers as 180 and 50 nm, respectively.

At first, the number of photons absorbed in the photoactive layer of the BHJ photovoltaic device is computed with an aid of the software FDTD Solutions, while the set of required optical parameters of the materials such as the relevant absorption coefficient are obtained from [21], and those for the sunlight spectrum from ASTM [22]. As an optical spacer layer, a zinc oxide (ZnO) which is a transparent electron acceptor with the refractive index of about 1.4 at 550 nm is chosen despite the fact that its relevant refractive index may vary with the ZnO coating method [23,24].

We scan the thicknesses of both the photoactive layer and the ZnO layer from 5 to 400 nm with a 5 nm step for the calculation of the absorbed photon number per unit area per unit time, i.e., *N*_*p*_ at the photoactive layer. This produces data of a 80-by-80 matrix form for the three-dimensional graph of *N*_*p*_ (*T*_*a*_,*T*_*s*_), where *T*_*a*_ and *T*_*s*_ are the thicknesses of the photoactive layer and the optical spacer, respectively. The absorbed photon number flux per unit time, which is denoted by *N*_*p*_, can be computed by integration over the standard solar irradiance spectrum (AM1.5 global) [20], as given by

(1) $$N_{p}=\int_{\lambda_{1}}^{\lambda_{2}}\frac{\Gamma \left(\lambda \right)}{E_{s}\left(\lambda \right)}\left(T_{t}-T_{b}\right)\mathrm{d\lambda}$$

where Γ(*λ*) is the solar irradiance spectrum as a function of wavelengths *λ*, *λ*_1_, and *λ*_2_ are the lower and upper limits of wavelengths for integration, *T*_*t*_ and *T*_*b*_ are the transmissions at the top and bottom of the active layer, respectively, and *E*_*s*_(*λ*) is the single photon energy at a given wavelength. Then, for a given quantum efficiency (*QE*), the short-circuit current (*J*_*sc*_) is [25]

(2) $$J_{\mathrm{sc}}=N_{p}\cdot \mathrm{QE}\cdot e_{0}\text{,}$$

where e_0_ is the elementary charge, e_0_ = 1.6 × 10^−19^C and *QE* is assumed to be 100%.

## Results and discussion

**Figure 2:**
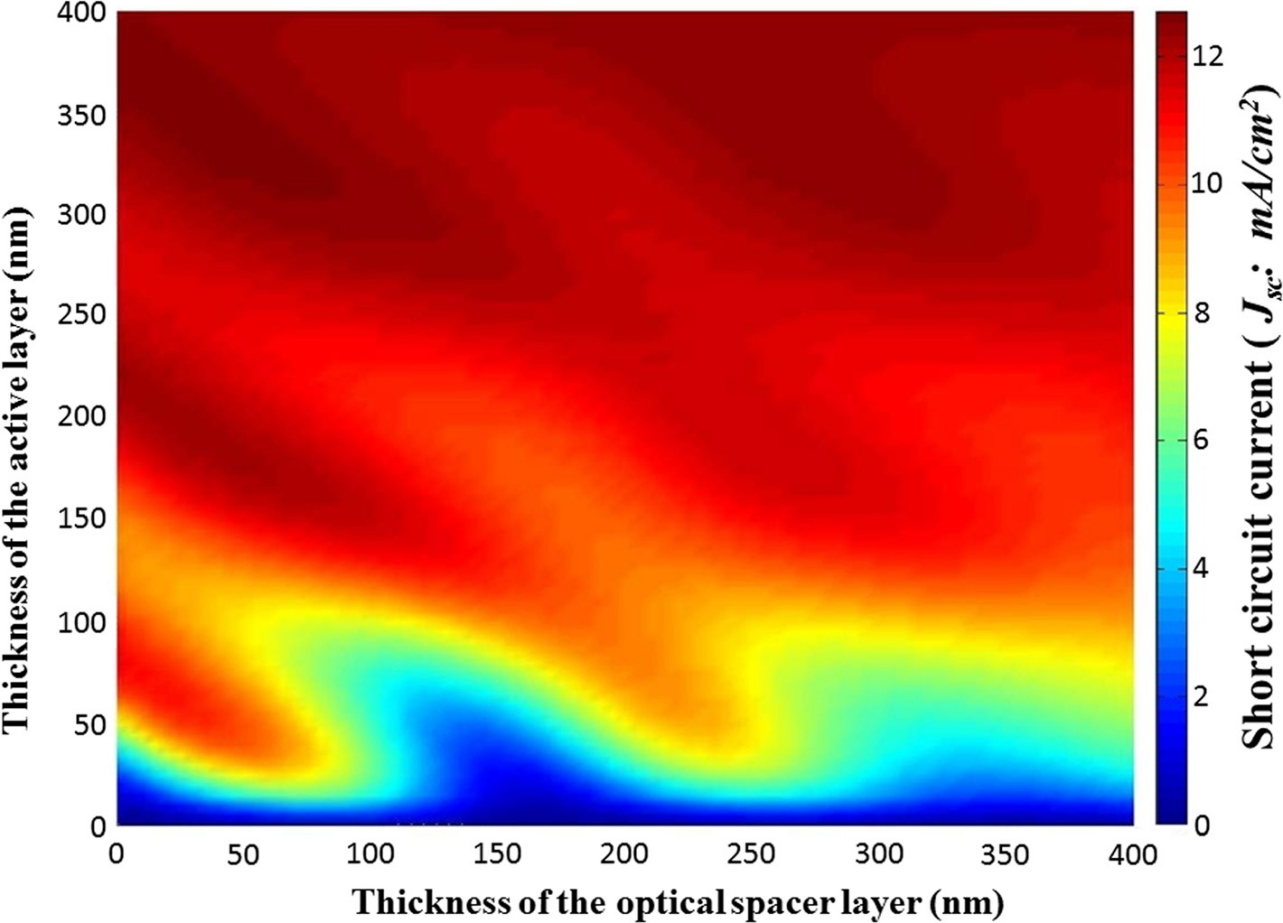
**Short-circuit current (*J***_***sc***_**) as a function of thicknesses of both layers, the photoactive layer and the optical spacer (ZnO).**

Figure 2 shows the calculated *J*_*sc*_ as the thicknesses of both the photoactive layer and the spacer are scanned. The fact that *J*_*sc*_ is in proportion to the intensity of the field within the photoactive layer reflects that *J*_*sc*_ can be modulated by thickness modulation of both layers through the light intensity redistribution induced by the effective optical path-length changes.

In the absence of a ZnO layer, increasing the photoactive layer thickness from 0 to about 80 nm increases *J*_*sc*_. The introduction of the spacer layer, however, causes this initial increase to occur with a higher slope for larger thickness of a ZnO layer (in the ZnO thickness range of 0 to 85 nm) as a consequence of the optical field intensity redistribution at the photoactive layer, due to the optical path-length change of the reflected light from the electrode. This causes the feature of the improvement of *J*_*sc*_ for the thin photoactive layer by inserting the thin ZnO layer, which is similar to [14]. For example, decreasing the active layer from 75 to 40 nm produces the drop in *J*_*sc*_ by only 8.4% upon the insertion of ZnO layer of 50-nm thickness. This is due to the ZnO-induced change in the interference of incoming photons with the photons reflected from the electrode. However, the slope at which the *J*_*sc*_ increases with increasing the photoactive layer thickness becomes small as the ZnO thickness exceeds about 100 nm as shown in Figure 2. This indicates that for a ZnO layer of thickness ranging from about 100 to about 200 nm, the increase of photoactive layer from 0 to about 100 nm benefits no substantial increase in *J*_*sc*_.

**Figure 3:**
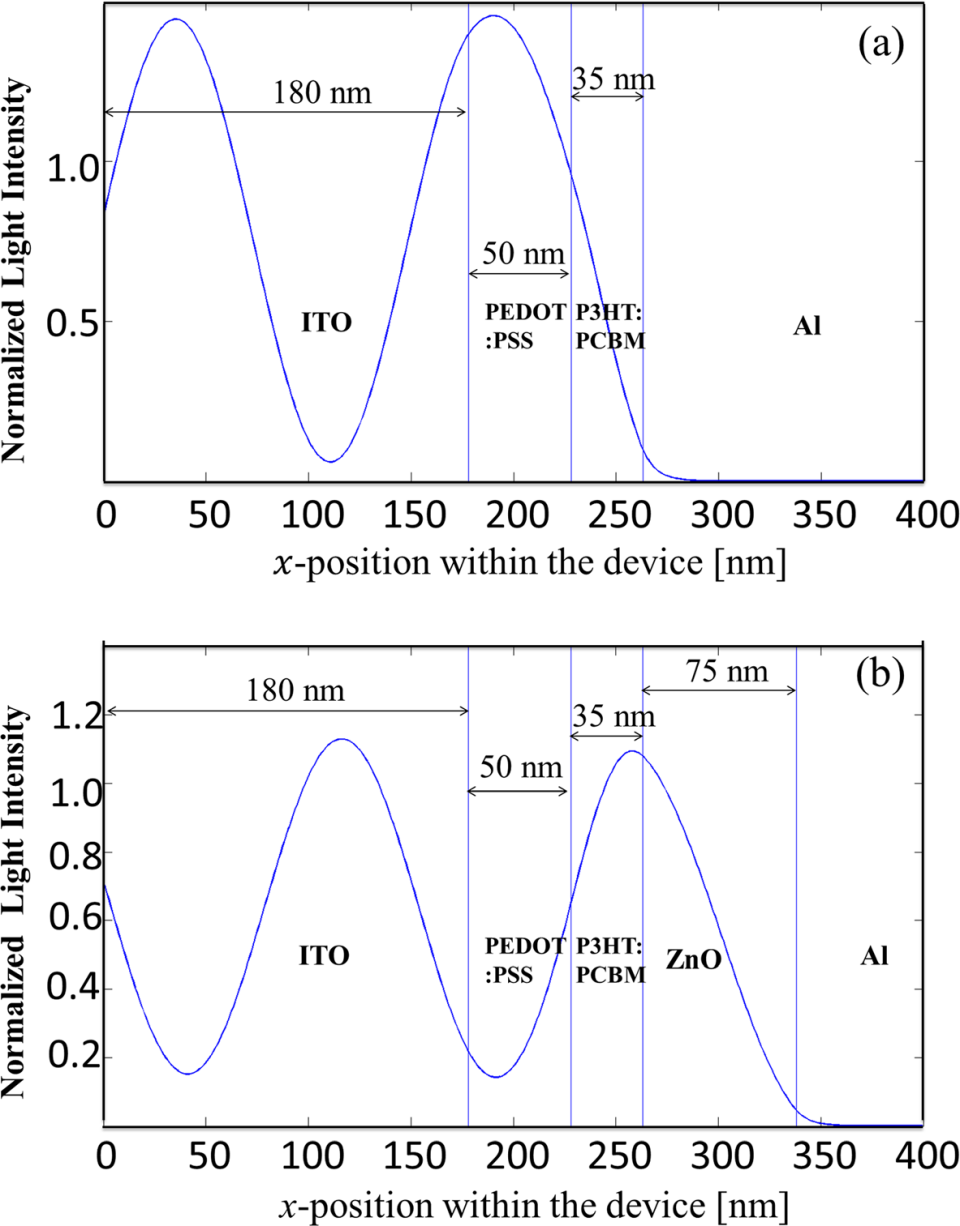
**Light intensity profile along the *x*-direction in the photovoltaic device.** Without **(a)** and with **(b)** the ZnO optical spacer inserted.

Figure 3a,b shows the electric field intensity profile across the *x*-position within the device without and with the ZnO layer of 75-nm thickness, respectively. It is visible that the insertion of a ZnO optical spacer of a proper thickness improves the light intensity at the photoactive layer, benefitting absorption of light, therefore, leading to more efficient harvest of light (Table 1).

**Table 1:** **Complex refractive indices at *λ*** **= 550 nm for each layer in the transfer matrix method calculation**

	**Number**	***k***
Glass	1.4516	0
ITO	1.8234	0.009
PEDOT:PSS	1.4871	0.03
P3HT:PCBM	2.1944	0.874
Optical spacer	1.4	0
Aluminum	0.96226	6.68255

The modulation behavior as shown in Figure 2 becomes faded out as the photoactive layer thickness exceeds about 300 nm, implying expectation of no further significant enhancement of *J*_*sc*_ by means of modulation of a ZnO spacer thickness. For the study of an optical spacer layer of a higher refractive index, we replace the ZnO spacer by TiO_2_ which has the refractive index of 2.0 at the visible light. We find that the *J*_*sc*_ modulates more rapidly with respect to the thicknesses in the three-dimensional graph (not shown) although the modulation patterns appear similar to the ZnO case. This suggests that the TiO_2_ thickness adjustment for the *J*_*sc*_ improvement (for a given thickness of the photoactive layer) requires the finer tuning, leading to more difficulty in fabrication of the spacer of such a fine-tuned thickness than the case of a ZnO layer.

It is known that the photoactive layer, i.e., a kind of the semiconducting layer, is the most resistant to charge transport among the other layers of the device. The P3HT:PCBM has the resistivity of about 10^3^ Ω < cm [26] whereas that of a ZnO can be adjusted below 1 Ω < cm by solution-based method [27] or even below 10^−3^ Ω < cm by CVD or PVD deposition methods [28]. The electrical resistance relevant to the active layer, thereby, must be taken into account in the calculation of the device efficiency. It is revealed that the device resistance is governed by the series resistance consisting of both the active layer bulk resistance and the contact resistance between the active layer and the Al electrode [15]. It is also known that the whole device resistance is approximately in proportion to the thickness of the photoactive layer, and reducing the resistance leads to higher PCE in organic photovoltaic devices. We assume the device is annealed in order to decrease the series resistance and can thus adopt its relation from [16], which represents the linear relationship between the thickness of the active layer (*T*_*a*_) and the series resistance (*R*_*s*_) for the annealed device, as given by

(3) $$R_{s}=0.06<T_{a}+3.1\text{.}$$

**Figure 4:**
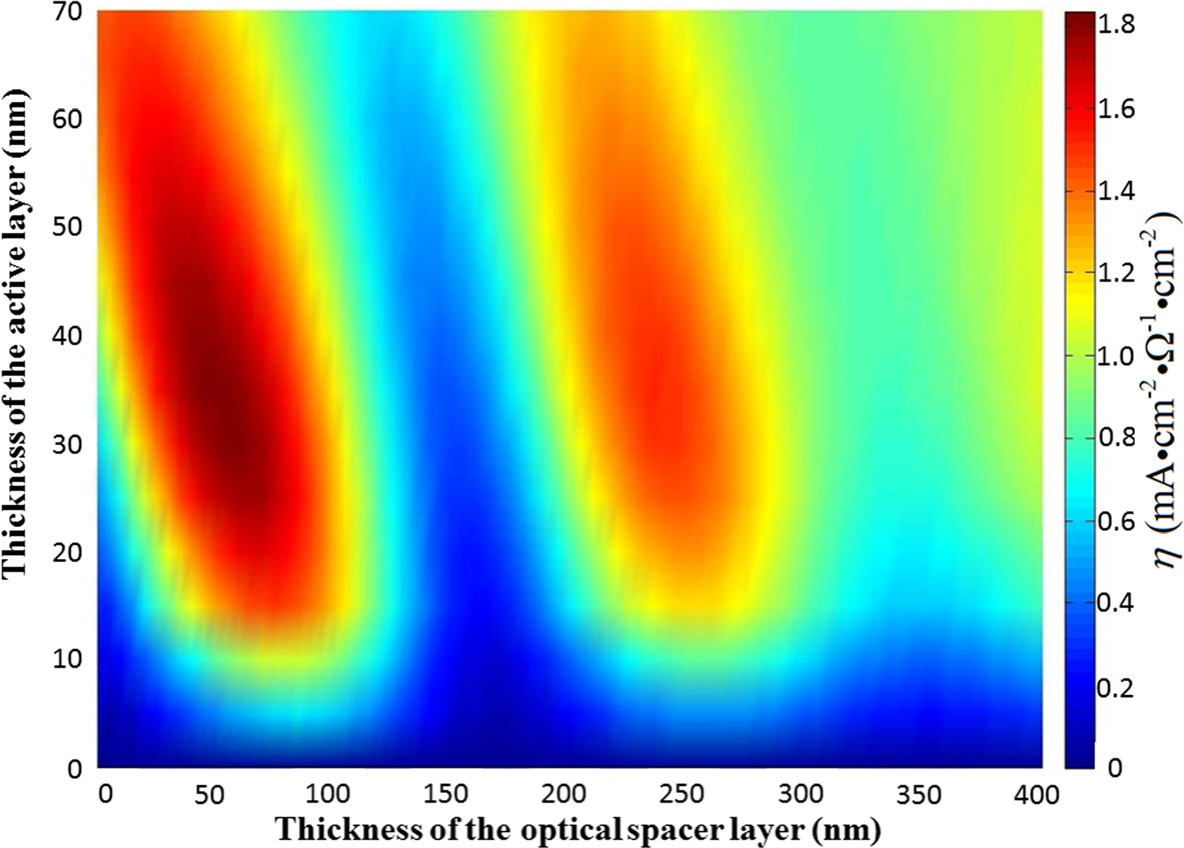
***η*** **=** ***J***_***sc***_**/*R***_***s ***_**as a function of thickness of both layers, the photoactive layer and the optical spacer (ZnO).**

This series resistance is used to normalize *J*_*sc*_ so that we can gain the estimation of the normalized number of photons absorbed per unit time per unit cross-sectional area of the device, for the device performance, i.e., *η* = *J*_*sc*_/*R*_*s*_. Figure 4 shows *η* (in the unit of mA · cm^− 2^ · Ω^− 1^ · cm^− 2^) in a top view of the 3D plot, which is achieved by scanning both the active layer thickness from 0 to 70 nm and the ZnO spacer thickness from 0 to 400 nm. The maximum *η* that accounts for the most effective device performance (*η* = 1.145 × 10^20^ mA · cm^− 2^ · Ω^− 1^ · cm^− 2^, *J*_*sc*_ = 10.5 mA · cm^− 2^) occurs at *T*_*a*_ = 35 nm and *T*_*s*_ = 50 nm, while the second maximum occurring at *T*_*a*_ = 35 nm and *T*_*s*_ = 235 nm.

The characteristics of the monotonic increase in *J*_*sc*_ in the range of 0 to 70 nm with the spacer thickness smaller than 50 nm, shown in Figure 2, are not visible in the behavior of the *η*, since the *η* shows the maximized value at the active layer thickness of 35 nm. This indicates that the series resistance as a function of active layer thickness will influence, qualitatively, the dependence of the device efficiency on thicknesses of both layers (the photoactive layer and the spacer layer). We also find that minimization of series resistance, e.g., via annealing methods, permits not only the relevant voltage to increase but also the optimum thickness of the photoactive layer to increase, leading to more absorption that can occur.

## Conclusions

We present, by simulation, the number of photons absorbed in the photoactive layer of the BHJ (P3HT:PCBM)-based organic photovoltaic device, in the case of using an optical spacer inserted between the photoactive layer and the electron collecting electrode.

The adjustment of an optical spacer thickness leads to the redistribution of optical intensity of light across the photoactive layer, enabling the maximization of the optical intensity for light absorption maximization, via controlling optical interference between the forward and backward light within the active layer. This interference modulation can be achieved by changing the effective optical path length of light propagating back and forth between the two electrodes, i.e., by changing the thickness of both the photoactive and spacer layers. We provide the 3D plots of the number of photons absorbed per unit time per unit area, as a function of thicknesses of both layers in the range of 0 to 400 nm. We also investigate the effect of using the different index spacer, e.g., the higher index layer of TiO_2_, and find that more delicate tuning of the spacer thickness is required in the case of the higher index spacer, for the device efficiency optimization.

In addition, series resistance, i.e., *R*_*s*_, composed of the active layer bulk resistance and its contact resistance with the electrode is taken into account to provide the three-dimensional plot of *η* = *N*_*p*_/*R*_*s*_. The results calculated show that the series resistance that depends on the photoactive layer thickness influences, qualitatively, the dependence of PCE on the thicknesses of both layers. This reveals that the proper annealing for elimination of such thickness-dependent resistance may be vital to increase not only the relevant voltage but also the optimum thickness of the photoactive layer.

Future works can include the dispersion of refractive index and absorption in the constituent layers such as a photoactive layer and a spacer for the calculation of the device absorption efficiency.

## Competing interests

The authors declare that they have no competing interests.

## Authors’ contributions

QL performed the simulation, analysis, and wrote the manuscript. WJY contributed to the completion of analysis used in the manuscript. HJ did the setup of the relevant research project for this manuscript, manuscript writing, and analysis. All authors read and approved the final manuscript.
